# The Moderating Role of Learning Rounds: Effects on Retrieval Practice and Context Dependence in Digital Flashcard Foreign Language Vocabulary Learning

**DOI:** 10.3390/bs15111540

**Published:** 2025-11-12

**Authors:** Shan Huang, Hong-Wen Cao, Jiayi Wang, Shutong Liu

**Affiliations:** 1 Research Center for Language, Cognition and Language Application, Chongqing University, Chongqing 401331, China; huangshan@stu.cqu.edu.cn; 2 School of Foreign Languages and Cultures, Chongqing University, Chongqing 401331, China; 3 College of Foreign Languages, Hunan Institute of Engineering, Xiangtan 411104, China; 14022@hnie.edu.cn; 4 Department of Radiology and Biomedical Imaging, University of California, San Francisco, CA 94607, USA; shutong.liu@ucsf.edu

**Keywords:** digital flashcards, foreign language vocabulary learning, learning rounds, retrieval practice effect, test modality

## Abstract

Digital flashcards are widely used in foreign language vocabulary learning. Research attributes their effectiveness to retrieval practice, where learners actively recall target information, thereby enhancing learning performance. The superiority of retrieval practice over non-retrieval methods such as restudying is referred to as the retrieval practice effect (RPE). Despite extensive research on the RPE, two key questions remain underexplored: how the number of learning rounds modulates the RPE in digital flashcard learning and whether the benefits of retrieval practice are consistently observed across different test modalities, a factor related to the context-dependence of memory. To address these questions, 108 Chinese–English bilinguals learned 60 Swahili–Chinese word pairs using two study methods (retrieval practice vs. restudying) under 3- or 4-round learning conditions, followed by cued-recall tests administered 30 min after learning, using both paper- and computer-based formats. Results showed that the RPE increased with more learning rounds. Additionally, retrieval practice consistently outperformed restudying method across different test modalities. These findings underscore the robustness of retrieval-based digital flashcard learning and provide practical insights for optimizing vocabulary instruction through strategic repetition scheduling.

## 1. Introduction

Vocabulary learning is a crucial aspect of foreign language (FL) learning, essential for reading comprehension (Laufer, 1992, 1997; Nation, 2011) and communicative competence (De Groot & Van Hell, 2005). Thus, developing a robust vocabulary is a primary concern for both FL teachers and learners. While mastering the entire vocabulary of a FL is unattainable, research has shown that knowing a core set of words suffices to establish linguistic competence in a FL for adults with secondary education (Hazenberg & Hulstun, 1996; Laufer, 1992; Nation, 2011). These findings suggest that intentional vocabulary learning may offer a viable method for building this foundational lexicon at the beginner stage (De Groot & Van Hell, 2005). Therefore, understanding which intentional learning methods most effectively promote durable vocabulary knowledge has become an important focus of FL acquisition research.

Given this importance, researchers have explored various approaches to facilitate intentional vocabulary learning, which is defined as “completing activities that are designed to promote word learning” (Webb et al., 2020, p. 716). Based on this definition, a meta-analysis of Webb et al. (2020) examined the effectiveness of four intentional activities, including flashcards, word lists, writing, and fill-in-the-blanks, for second language (L2) vocabulary acquisition and reported that flashcard learning was the more effective approach for strengthening form–meaning connections. In a typical flashcard learning procedure, a set of cards displays a foreign word on one side and its translation in the learners’ native language on the other side. Learners first view the foreign word and attempt to recall its meaning and then flip the card to verify their answer (Nakata, 2019). This method exemplifies retrieval practice, where the foreign word acts as a retrieval cue, prompting learners to search their memory for the target information, while the native translation provides corrective feedback, allowing learners to evaluate, revise, and reinforce the form–meaning association of each word pair. Such retrieval-based processing has been identified as a central mechanism underlying the effectiveness of flashcard learning (Lei & Reynolds, 2022; Nakata, 2019).

Extensive research demonstrated that learning activities involving active retrieval produce better learning outcomes than non-retrieval activities, a phenomenon known as the retrieval practice effect (RPE) (Adesope et al., 2017; Karpicke, 2017; Roediger & Butler, 2011; Roediger & Karpicke, 2006b; Rowland, 2014). Studies on the mechanisms of flashcards in FL vocabulary learning consistently found an advantage over passive word-list learning that relies on mere repetition of word pairs (Kanayama & Kasahara, 2016; Li et al., 2024; G. S. Van den Broek et al., 2014). These studies reveal the RPEs and provide evidence for the beneficial effects of active retrieval in flashcard learning.

While flashcard learning benefits primarily from memory retrieval, the magnitude of the RPE may be moderated by the number of learning repetition, which is a well-established factor crucial for effective vocabulary acquisition (Uchihara et al., 2019; Webb, 2007) and demonstrated positive effects in both repeated study and repeated retrieval conditions (Karpicke & Roediger, 2007, 2008; Nakata, 2017). For instance, Karpicke and Roediger (2008) conducted a study comparing four learning conditions: study–test, repeated study only, repeated test only, and neither study nor test after initial learning. The results showed that as the cumulative proportion of recalled word pairs increased, learners nearly fully mastered all target words, and there were no significant differences in the learning curves across the four conditions. These findings indicated that with an increase in the cumulative recall of word pairs, learners nearly fully mastered all target words, showing no significant differences in the learning curves across four conditions. This suggested that adequate repetition, whether through restudy or retrieval practice, can substantially enhance vocabulary learning. Nevertheless, repetition does not invariably lead to an increase in the RPE, which signifies the additional memory advantage of retrieval practice over alternative approaches (typically restudy). If both approaches yield comparable benefits from additional learning opportunities, the RPE may not proportionately increase. Consequently, the impact of learning rounds on the RPE in flashcards learning remains unclear.

Furthermore, a fundamental goal of FL vocabulary learning is to facilitate the application of acquired words in various contexts. This goal makes it essential to understand how different learning methods affect the flexibility of memory representations. Previous research has shown that retrieval practice, as opposed to restudy, tends to produce more flexible and context-independent memory representations that can support transfer across context (Karpicke, 2017; Roediger & Butler, 2011). These findings suggest that active retrieval may reduce the reliance of memory representations on the encoding context, thereby enhancing retrieval flexibility in new situations. However, the findings of Halamish and Elias (2022) contradicted this notion. Their study, which manipulated the consistency of studying and testing media, revealed no significant performance gap between retrieval practice and restudy. According to the contextual variability hypothesis (Lohnas et al., 2011), this absence of the context-dependent differences between the two learning methods may result from insufficient learning opportunities. This inadequacy can impede effective contextual processing during retrieval practice, thus hindering a notable reduction in context-dependence. Therefore, further investigations are warranted to determine whether an increase in learning round would unveil these distinctions.

As the integration of digital technologies in education has popularized the use of digital flashcards (DFs) for FL vocabulary learning (Ji & Aziz, 2021; Lei & Reynolds, 2022; Lin & Lin, 2019; Nakata, 2011). Previous studies have shown that learning with DFs can yield outcomes comparable to or better than paper-based flashcards (PFs) in specific scenarios (Ashcroft et al., 2018; Dizon & Tang, 2017; Yüksel et al., 2022), highlighting their potential for investigating retrieval-based learning mechanisms. Therefore, the present study examined the effects of learning rounds via computer-based flashcards on the RPE and the context-dependence of memory in FL vocabulary learning. By exploring these aspects, the study provides empirical insights into the consistency of digital flashcard learning across contexts and offers practical implications for enhancing the design of DFs system through optimized learning rounds.

## 2. Literature Review

### 2.1. Digital Flashcards (DFs) in Foreign Language Vocabualry Learning

A common approach to evaluating the effectiveness of DFs in FL vocabulary learning involves comparing them with traditional learning methods, such as PFs and word lists (Ashcroft et al., 2018; Dizon & Tang, 2017; Najafi Karimi & Kheradmandi Amiri, 2025; Sage et al., 2020; Xodabande et al., 2022, 2023; Yüksel et al., 2022; Zarrati et al., 2024). The primary goal of this comparative research is to determine whether digital tools lead to superior learning outcomes. For example, Dizon and Tang (2017) compared the effects of PFs and DFs on receptive and productive vocabulary learning through a 12-week experiment. The results revealed that both learning methods significantly improved vocabulary performance, but the difference in learning outcomes between the two methods was not significant. Yüksel et al. (2022) conducted a 10-week experiment to examine the effects of DFs on technical vocabulary learning compared to word lists. Using a pre-and-post-test design, they found that students using DFs achieved greater vocabulary gains. While these studies validate the overall utility of DFs, their methodological design often limits deeper mechanistic insights. Both of these studies, like others in this vein, were implemented in the authentic instructional settings over extended periods. This characteristic, while ecologically valid, introduces a fundamental challenge for isolating the specific contribution of active retrieval, because it becomes difficult to ensure equivalent learning time or strict adherence to assigned procedures across conditions.

To isolate and measure the RPE within digital flashcard learning, a controlled laboratory paradigm has been established as the methodological standard (Candry et al., 2020; Kanayama & Kasahara, 2016; Li et al., 2022, 2024; G. S. Van den Broek et al., 2014, 2018). This approach adopts the classic three-phase retrieval practice paradigm (initial study, intervention with retrieval practice vs. restudy, and final test) to minimize confounding variables (Roediger & Karpicke, 2006a; G. Van den Broek et al., 2016). For example, Kanayama and Kasahara (2016) examined the RPE in digital flashcard L2 vocabulary learning using the classic paradigm. In the first phase, all participants initially studied the complete set of word pairs. Then, they proceeded to the second phase, where they were assigned to different learning interventions: the experimental group used DFs, while the control group studied from word lists. Participants in the DFs group studied by recalling the L1 translations corresponding to L2 cues and verifying their answers, a retrieval-based method. In contrast, learners in the word-list group engaged in repeated study of the intact word pairs, a method known as restudy. Results from the final phase showed that participants in the DFs condition outperformed those in the word-list condition on both immediate and one-week delayed cued recall tests, demonstrating robust RPEs. Building on this paradigm, the present study adopts its core procedural logic, including the phased structure and the critical comparison between retrieval-based and restudy-based interventions, to ensure a valid assessment of the RPE in the current experimental context.

### 2.2. Factors Affecting the RPE in Digital Flashcard Foreign Language Vocabulary Learning

Studies indicate that several factors that modulate the RPE in flashcard learning, with retrieval intervals and provision of feedback being two of the most critical (Nakata, 2019; Roediger & Butler, 2011). In a series of experiments, Pyc and Rawson (2009) had participants practice retrieving the English equivalents of 70 Swahili–English word pairs, with the interval between retrieval attempts manipulated to be either 1 or 6 min. Their results showed that longer retrieval intervals produced better delayed recall than shorter intervals, suggesting that the effectiveness of retrieval practice improves with increased spacing. Regarding feedback, Butler and Roediger (2008) examined the impact of feedback on this effect in passage learning context under three conditions (no feedback vs. immediate feedback vs. delayed feedback) during the learning phase. The results demonstrated that learners in both feedback conditions significantly outperformed those who engaged in retrieval practice without feedback. Consistent with the study by Li et al. (2024), the RPE in third language (L3) vocabulary learning was observed only when retrieval practice was coupled with corrective feedback. Prior research indicates that when a retrieval attempt fails without corrective feedback, learners lack access to the correct information, consequently undermining the benefits of retrieval practice (Kang et al., 2007). Conversely, when corrective feedback is provided, it not only facilitates error correction (Pashler et al., 2005) but also increases the likelihood of subsequent retrieval success (Butler et al., 2008), thereby solidifying the benefits of retrieval practice.

Accordingly, the present study was designed to incorporate these empirical insights. First, immediate corrective feedback was included in the retrieval practice condition, as it is inherent to the DFs and is crucial for enhancing the RPE. Second, given the established advantage of spaced retrieval, the interval between retrieval attempts was extended by structuring the learning session into multiple rounds, with each round comprising a full cycle through all word pairs. Finally, to preclude the potential confound of unequal learning time, the total study duration was strictly matched between the retrieval and restudy conditions.

### 2.3. Predictions of the Moderating Effect of Learning Round on the RPE

Theoretical frameworks concerning the underlying mechanisms of the RPE lead to divergent predictions regarding the moderating effect of learning rounds. According to the retrieval effort hypothesis, the mnemonic benefits of retrieval practice arise from the cognitive effort involved in retrieving information from memory. Specifically, higher cognitive effort during active retrieval produces greater learning gains, whereas when retrieval becomes easier and cognitive effort declines, the benefits of retrieval practice may diminish (Glover, 1989; Karpicke & Roediger, 2007; Pyc & Rawson, 2009). For example, Pyc and Rawson (2009) provided empirical support for this idea by manipulating the interstimulus interval (ISI) and the criterion level during learning phase. They found that longer ISIs, which increased retrieval difficulty, led to better performance, while higher criterion levels, which made retrieval easier, resulted in diminishing returns in delayed recall performance. These findings suggest that the benefit of retrieval practice depends on cognitive effort, implying that over multiple learning rounds, the marginal gain of each additional round may decrease as retrieval becomes more automatic.

In contrast, the elaborative retrieval hypothesis (Carpenter, 2009) offers a different perspective on the role of repeated practice. This framework conceptualizes retrieval as an active, reconstructive process where learners activate sematic information associated with the cue word and integrate it with the target during successful recall (Carpenter & Yeung, 2017). This process results in a more elaborate memory representation. With each successful retrieval, this integrative processing is believed to accumulate, progressively strengthening and consolidating the memory trace. It follows that increasing retrieval frequency should continuously enhance learning outcomes. Empirical support for this view comes from Nakata (2017), who investigated the effects of within-session retrieval frequency on L2 vocabulary learning. In his study, conditions with five and seven retrieval opportunities led to significantly higher posttest scores than those with only one or three attempts. This pattern of cumulative benefits supports the hypothesis, suggesting that more frequent retrieval provides greater opportunity for elaboration and consolidation. Consequently, from this theoretical standpoint, increasing the number of learning rounds should lead to sustained, and potentially even growing benefits for retrieval-based learning.

The aforementioned theories offer different predictions for retrieval practice: its benefits may either diminish if the process becomes automated or increase if elaborated retrieval dominates with more learning rounds. In contrast, the prediction for restudy is more straightforward, as increased learning opportunities constantly enhance performance by enriching lexical representations through repeated exposure (Horst, 2013). Therefore, this study predicts that the moderating effect of learning rounds on the RPE would be determined by the dynamic interplay between these two learning trajectories: an increasing RPE would support the elaborative retrieval account, indicating that additional rounds continue to benefit retrieval practice more than restudy, while a stable or diminishing RPE would indicate that the benefits of retrieval practice are constrained, consistent with the retrieval effort hypothesis.

### 2.4. Predicting the Moderating Role of Learning Rounds on Memory Context-Dependence

The contextual variability hypothesis proposes that each learning or retrieval episode is encoded and bound to a specific contextual state, which integrates external sensory input with internal cognitive conditions (Estes, 1955; as cited in Lohnas et al., 2011). As these contextual representations gradually diverge over time with shifts in mental states or external environments, memories become associated with a broader set of contextual cues, thereby supporting flexible retrieval. Applied to learning, repeated recall promotes the re-encoding of memories across multiple retrieval attempts, each of which occurs in a slightly different contextual state. This process forms a rich associative network by linking the memory to a diverse set of cues, thus enhancing its flexibility. In contrast, repeated study primarily reinforces the original item-context associations under highly similar conditions. This process produces a relatively unitary and context-specific memory traces, which is more susceptible to performance decrements when the retrieval context changes.

This contrast leads to memory representations with different level of flexibility, and empirical research controlling for test formats has supported this inference (Butler, 2010; Pan et al., 2015; Whiffen & Karpicke, 2017). For instance, Whiffen and Karpicke (2017) conducted three experiments to test the episodic context account of retrieval practice. After studying word lists, participants either restudied the words or performed list discrimination judgments, requiring them to retrieve the original episodic context and thus repeatedly recall the items across varying contexts. Results showed that, compared with restudy, retrieval practice significantly enhanced free recall and improved temporal organization, supporting the view that repeated retrieval across multiple contexts fosters more flexible memory representations. However, Halamish and Elias (2022) found no differences in memory flexibility between the two learning methods when the learning and testing contexts were mismatched, which is inconsistent with the prediction made by the contextual variability hypothesis.

One possible explanation is that changing only the testing medium while keeping the cognitive task identical provides limited contextual variability. Based on the contextual variability hypothesis, retrieval practice enhances memory flexibility by associating information with diverse contextual cues, but if cognitive operations are the same, opportunities to form distinct contextual representations are minimal, and the advantage of retrieval practice may not emerge. Another potential factor is insufficient learning opportunities. When retrieval attempts are limited, memory traces may not be rich enough to reveal flexibility differences. The present study therefore hypothesizes that increasing the number of learning rounds will distinguish these competing accounts: a persistent absence of differences would implicate limited contextual variation, whereas the emergence of differences would instead indicate that prior null findings were due to insufficient learning opportunities.

### 2.5. Present Study

The present study aims to investigate the moderating role of learning rounds on the RPE and the context-dependence of memory representations in digital flashcard learning. The study used a design in which learning rounds (three vs. four) functioned as a between-subjects variable, while learning method and test modality were manipulated as within-subjects variables. Building on prior studies (Kanayama & Kasahara, 2016; Li et al., 2022, 2024), a classic retrieval practice paradigm was employed to investigate the RPE. In the experimental condition, computer-based flashcards were used to induce retrieval practice, while the control condition involved computer-based restudying of word lists. The learning procedure was closely monitored in the laboratory to ensure adherence to the prescribed learning methods. Learning outcomes were assessed via a cued recall test, and two types of test modalities (paper-based vs. computer-based) were employed.

To optimize experimental control, the retrieval task in both learning and testing phases required learners to recall the meanings of foreign words rather than their forms. This design avoided the difficulties inherent in word-form production, which depends heavily on accurate orthographic knowledge and is easily affected by interference from the learner’s native language (Nation, 2001; as cited in Mondria & Wiersma, 2004). Such factors could have otherwise introduced individual differences in productive ability, thereby confounding the results. By minimizing these sources of variability, the meaning-recall task offered a clearer assessment of how the number of learning rounds influenced the retrieval practice effect and strengthened the internal validity of the experiment.

To ensure that observed differences in memory performance reflect the intrinsic cognitive processing distinctions between restudy and retrieval practice, rather than extraneous factors, this study implemented strict controls to establish equivalence between computer-based and paper-based testing. Research on test media has indicated that computer-based tests, due to their multimodal (Cordero et al., 2018; Wang et al., 2008) and interactive features (Chua & Don, 2013; Singleton, 2001), can enhance learner engagement (Dujardin et al., 2021). If uncontrolled, however, these medium-specific characteristics may introduce systematic bias and confound performance interpretation. Importantly, Shahini and Hashemi Toroujeni (2023) demonstrated that, under strictly standardized testing conditions, such biases can be effectively controlled, with computer- and paper-based tests achieving comparable reliability and score distributions. Building on these insights, the present study adopted non-interactive DFs combined with predetermined, non-contingent corrective feedback, which remained the same regardless of learners’ responses. This design minimized procedural and multimodal differences, ensuring equivalence in core cognitive processing, and thereby allowing any observed performance differences to more reliably reflect the cognitive mechanisms underlying the learning phase.

To examine how learning rounds moderate the advantage of retrieval practice over restudy, recall performance was compared between two learning methods. A significant interaction between learning rounds and learning method would indicate that the magnitude of the RPE varies depending on the number of practice opportunities, whereas a non-significant interaction would suggest that learning rounds do not meaningfully influence the retrieval advantage. To explore how learning rounds influence the reliance of retrieval practice and restudy memories on the testing context, the context-dependence of memory representations was operationalized as the difference in recall performance between the test modalities for each learning method. A significant interaction among learning round, learning method, and test modality would indicate that the effect of testing context varies depending on the number of learning rounds, whereas a non-significant interaction would suggest that learning rounds do not meaningfully alter context-dependence.

The research questions are as follows:

(1) To what extent do different learning rounds (e.g., three vs. four) moderate the RPE in learning FL vocabulary through DFs?
(2) To what extent do different learning rounds (e.g., three vs. four) moderate the context-dependence of memory representations formed through two learning methods?

## 3. Method

### 3.1. Participants

To control for the baseline of participants’ language learning experience, this study recruited 108 Chinese-native speaking students (*range_age_* = 18–31; *M_age_* = 21.48; *SD_age_* = 2.83) with English as their L2. All participants had acquired their L2 through formal school education, with an average duration of English instruction in school of 11.75 years (*SD* = 2.58) for the 3-round group and 12.33 years (*SD* = 2.90) for the 4-round group, respectively. None of the participants had experience learning other FL, nor had they any experience studying or living abroad.

To ensure that participants in the two groups did not differ significantly in L2 proficiency, their English ability was assessed using a 7-point Likert self-rating scale (1 = very poor; 7 = native-like) and the LexTALE test (Lemhöfer & Broersma, 2012). The results in Table 1 indicated that participants were intermediate-level Chinese–English bilinguals. An independent-sample *t*-tests revealed no significant differences between the two groups in either subjective or objective proficiency scores (all *p*s > 0.05).

In addition, one pre-experiment survey (Supplementary Material S1) was administered to investigate participants’ perceived difficulty of L2 vocabulary learning using a 7-point Likert scale (1 = very easy; 7 = very difficult). The result revealed moderate perceived task difficulty, with a mean rating of 3.93 (*SD* = 1.08). And their prior experience with using electronic devices for FL vocabulary learning and testing was also explored. Over 90% of the participants reported having experience in using electronic devices for L2 vocabulary learning (such as using digital vocabulary books or flashcard applications), and more than 70% indicated that they had participated in L2 vocabulary tests conducted via electronic devices (such as online quizzes or computer-based assessments), confirming their general familiarity with the core learning and testing methods employed in this study. The survey also examined participants’ attitudes toward test modality. Over half of the participants (52.73%) believed that the test modality has an impact on their performance.

Although L2 learning experience and proficiency among participants may influence learning outcomes across experimental conditions, selecting this population is methodologically justified. This group represents one of the most common and representative profiles of FL learners in China. Such a sample population helps control for variability in L2 learning experience, educational background, and language ability, reducing the impact of individual differences on the observed results. Furthermore, it enhances the ecological validity of the study and strengthens its practical relevance. To mitigate the influence of participants’ language background on the findings, a mixed experimental design was adopted, with learning round as a between-subjects variable and learning method and test modality as within-subject variables.

### 3.2. Materials

This study selected Swahili–Chinese word pairs as learning material for several reasons. First, it is entirely unfamiliar to the participants, ensuring that prior exposure does not confound the observed learning effects. Second, unlike languages that employ non-Latin scripts (e.g., Thai or Arabic), Swahili is written in a Latin-based orthography consisting of 26 letters, identical to that of English. This orthographic correspondence minimizes the cognitive demands associated with processing unfamiliar scripts and allows Chinese–English bilingual participants to engage with the learning task more efficiently. Finally, Swahili has been widely used as learning material in studies on the RPE (Karpicke & Roediger, 2008; Pyc & Rawson, 2009; Wilkinson et al., 2019). These studies typically adopted or modified word pairs from the Swahili–English list developed by Nelson and Dunlosky (1994), using English as a mediating language to create versions such as Swahili–Swedish (Wiklund-Hörnqvist et al., 2022) or Swahili–Hebrew (Halamish & Elias, 2022) word pairs.

Following this convention, the present study developed 60 Swahili–Chinese word pairs (*range_letter_* = 4–7; *M* = 5.60, *SD* = 0.91) suitable for the current experiment. The corresponding Chinese words were translated from the Swahili–English word list (Nelson & Dunlosky, 1994) according to the 6th edition of *Oxford Advanced Learners’ English-Chinese Dictionary* (Hornby, 2004). All Chinese translations were selected from the most frequently used two-character nouns (*M _stroke_* = 16.58, *SD* = 5.15).

All word pairs were evenly divided into four sublists to control for potential influences of lexical characteristics on memory performance (Supplementary Material S2). Each sublist followed an identical composition pattern, containing 2 four-letter, 4 five-letter, 7 six-letter, and 2 seven-letter Swahili words, resulting in 15 pairs per list. In Table 2, the number of strokes in the corresponding Chinese translations was calculated and compared across the four sublists, with no significant differences observed, ensuring lexical equivalence among the materials (both *p*s > 0.05). To control for the potential confounding effects of word difficulty on memory performance (Vaughn et al., 2013), the present study used the recall accuracy rates of Swahili–English word pair after three cycles of learning and testing reported in Nelson and Dunlosky’s study (1994) as an operational indicator of item difficulty during the material grouping stage. Statistical analyses revealed no significant differences in recall accuracy among the four sublists (all *p*s > 0.05), indicating comparable difficulty levels across conditions (Table 2).

In addition, a counterbalanced list design was implemented to control for potential confounding effects of item characteristics and word difficulty (Supplementary Material S3). Specifically, each participant studied two sublists under the repeated study condition and the remaining two under the retrieval practice condition. The assignment of sublists to learning conditions was rotated across participants. This design ensured that each sublist appeared equally often in both learning conditions, enhancing the reliability and validity of the measured retrieval practice effect.

### 3.3. Procedure

**Figure 1:**
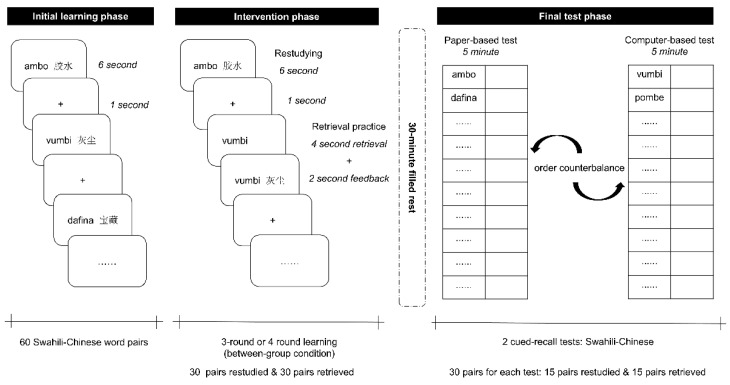
Schematic view of the experimental procedure. All word pairs were randomly displayed in the three phases, and the assignment of sublists to within-participants conditions was counterbalanced across participants.

The classic retrieval practice paradigm (Kanayama & Kasahara, 2016; Li et al., 2022, 2024; G. Van den Broek et al., 2016) used by this study was depicted in Figure 1. In the initial learning phase, 60 Swahili–Chinese word pairs were presented in the center of computer screen with a random order. Each word pair was presented for 6 s, followed by a crosshair of 1 s. Participants were instructed to learn and memorize these word pairs through silently reading. After learning 60 pairs, participants were asked to complete a 3 min distraction task which required them to continuously subtract 3 from 600 and orally report the results.

Following the distraction task, they came to the intervention phase. Participants in the 3-round learning group were required to undergo three rounds of the experimental intervention (i.e., 180 trials in total), while participants in the 4-round learning group would take the intervention four times (i.e., 240 trials). In each learning round, they had to learn 30 pairs through restudying and the other half through retrieval practice. Under the restudying condition, intact word pair was displayed in the center of the screen for 6 s. Participants were required to learn these pairs with the same manner as in the initial learning phase. Under the retrieval practice condition, Swahili word was presented first for 4 s and then the Chinese translation equivalent appeared for 2 s. Participants were instructed to recall Chinese words based on provided Swahili words, and then check their answers with the given corrective feedback. Four sublists were counterbalanced between study methods within groups, and all stimuli were presented in random order (Supplementary Material S3). Participants underwent a 30 min filler interval following the learning phase. This interval was designed to prevent participants from actively recalling the learned word pairs during the break, avoiding potential contamination of the effects of the two learning methods.

In the final test phase, participants were asked to recall corresponding Chinese translations based on these learned Swahili words, which were presented on either paper-based or digital answer sheets. For each learning method, one sublist was tested on paper and the other on a digital platform, with the assignment of sublists to test modality counterbalanced across participants. Under the paper-based test condition, participants were given 5 min to write their answers. In the computer-based condition, participants had 5 min to type their responses on a digital test sheet using Pinyin input. The digital test sheets were created and presented using Microsoft Word 2021. The order of two test versions was counterbalanced within groups and between participants.

### 3.4. Data Analysis

#### 3.4.1. Scoring

Strict scoring criteria were applied in this study. An answer was scored as correct (“1”) if it either exactly matched the Chinese translation provided on the answer sheet or was a synonymous expression conveying the same meaning. All other responses were scored as incorrect (“0”). Minor writing errors did not affect the determination of correctness. The recall accuracy was calculated as function of the four within-group conditions under two learning round conditions: restudying with paper-based test (RSP), restudying with computer-based test (RSC), retrieval practice with paper-based test (RPP), and retrieval practice with computer-based test (RPC).

#### 3.4.2. Statistical Analysis

The recall accuracy data were analyzed using a GLMM with a binomial distribution and a logit link function. The analysis was conducted in R Studio 4.3.1 (R Core Team, 2023) using the *glmer* function from the *lme4* package (Jaeger, 2008). *p*-values for the fixed effects were obtained using the *lmerTest* package (Kuznetsova et al., 2017). The binary dependent variable was the recall accuracy (correct/incorrect, coded as 1/0). The fixed effects included study method, test medium, learning round, and all their possible two-way and three-way interactions. The random-effects structure included random intercepts for both participants and items in order to account for variability arising from these sources and to obtain more precise estimates of the experimental effects.

Model fitting employed a backward elimination approach, guided by the principle that comparing nested models is a robust method for identifying significant effects (Barr et al., 2013). Specifically, the significance of main effects and interactions was assessed by comparing a full model against a series of reduced models, each lacking a specific effect of interest (e.g., the three-way interaction, a two-way interaction, or a main effect), using likelihood ratio tests (LRT). A significant result (*p* < 0.05) indicated that the model including the effect provided a significantly better fit to the data.

For significant effects, post hoc analyses were performed using the *emmeans* package (Lenth et al., 2021) to estimate marginal means and conduct pairwise comparisons. The results of these comparisons are reported as odds ratios (OR) with 95% confidence intervals, and *p*-values were adjusted using the Bonferroni method to control for multiple comparisons.

## 4. Results

The descriptive statistics for recall accuracy across all conditions are summarized in Table 3. Overall, the results reveal three key patterns. First, retrieval practice consistently yielded superior recall accuracy compared to restudying, regardless of the testing modality. Second, recall accuracy remained comparable between the two testing modalities for both learning methods. Most importantly, four learning rounds provided a clear additional benefit over fewer rounds under all experimental conditions.

**Figure 2:**
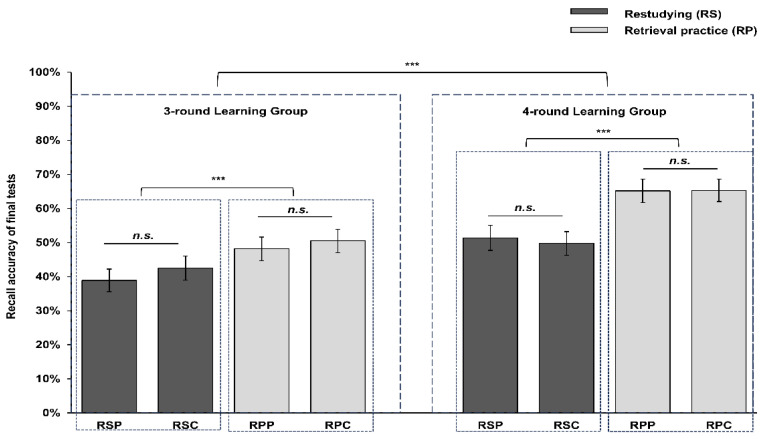
Mean recall accuracy of final tests for each study method, test medium and learning round. Error bars represent standard errors of the mean. RSP refers to restudying with paper-based test. RSC refers to restudying with computer-based test. RPP refers to retrieval practice with paper-based test. RPC refers to retrieval practice with computer-based test. “***” represents *p* < 0.001. “*n.s.*” represents non-significant.

As shown in Figure 2, overall model results indicated that the three-way interaction among study method, learning round, and test medium was not significant (*χ*^2^ (1) = 0.65, *p* = 0.42), suggesting that the effect of retrieval practice across learning rounds did not differ between test modalities. Main effects revealed that study method was significant (*χ*^2^ (4) = 159.41, *p* < 0.001), and learning round was also significant (*χ*^2^ (4) = 19.20, *p* = 0.0007). Test medium showed no significant main effect (*χ*^2^ (4) = 2.72, *p* = 0.61). Among the two-way interactions, only the study method × learning round interaction reached significance (*χ*^2^ (1) = 7.97, *p* = 0.0048), indicating that the retrieval practice effect varied across learning rounds, whereas the interaction of study method and test medium (*χ*^2^ (1) = 0.18, *p* = 0.67) or test medium × learning round (*χ*^2^ (1) = 1.33, *p* = 0.25) was not significant.

To probe the significant interaction between study method and learning round, we conducted pairwise comparisons using *emmeans*. Results showed that under 3-round learning condition, retrieval practice (RT) yielded higher accuracy than restudy (RS) with a probability of 0.50 vs. 0.354, odds ratio (OR) = 0.547, SE = 0.0486, z = −6.784, *p* < 0.0001, 95% CI [0.381, 0.620]. In 4-round learning condition, the advantage recall accuracy of retrieval practice was even greater, with RT probability = 0.739 vs. RS = 0.519, OR = 0.382, SE = 0.0349, z = −10.524, *p* < 0.0001, 95% CI [0.634, 0.822]. Examining the effect of learning rounds within each study method, RS improved from 0.354 to 0.519 with the increase in learning rounds (OR = 0.507, SE = 0.144, z = −2.386, *p* = 0.017, 95% CI [0.251, 0.472]), whereas RT improved from 0.500 to 0.739 (OR = 0.354, SE = 0.101, z = −3.648, *p* = 0.0003, 95% CI [0.381, 0.620]). These results indicate that the retrieval practice effect is enhanced by additional learning rounds, and this moderating effect is consistent across test modalities, as no interaction with test medium was observed.

## 5. Discussion

In the current study, we aim to explore how repeated learning opportunities affect the RPE and the extent to which memory representations are context-dependent in digital flashcard learning. The major findings of this study are that the RPE tends to increase with additional learning opportunities, and that the effect of learning method is consistent across different test modalities and remains stable regardless of the number of learning rounds.

Consistent with previous research (Halamish & Elias, 2022; Kanayama & Kasahara, 2016; Li et al., 2024), the present study found significant RPE in digital flashcard FL vocabulary learning under both low (3-round)- and high (4-round)-learning-opportunity conditions, indicating that the advantage of retrieval practice combined with corrective feedback is relatively robust. Moreover, prior studies have shown that the benefits of retrieval practice increase with retrieval frequency (Karpicke & Roediger, 2008; Nakata, 2017). For example, Nakata (2017) investigated the effects of within-session retrieval frequency on L2 vocabulary learning and found that performance increased with retrieval frequency, with five and seven retrievals producing the highest scores, indicating cumulative memory gains from repeated retrieval. The present findings align with this pattern: learners’ performance in the retrieval practice condition improved with additional learning opportunities. While performance improved in both conditions, the gains were more pronounced in the retrieval practice group. This led to an amplification of the RPE over successive rounds, revealing its cumulative and dynamic nature. These results support the predictions of the elaborative retrieval hypothesis (Carpenter, 2009), which suggests that repeated retrieval enhances memory representations by activating related semantic concepts and forming interconnected knowledge networks. Through repeated practice, learners may progressively expand the semantic cue-target associations in each round and gradually strengthen overall semantic networks, thereby expanding the advantage of retrieval practice.

Although the present findings seem inconsistent with the predictions of retrieval effort hypothesis, this inconsistency should be interpreted with caution. According to this hypothesis, the mnemonic benefits of retrieval practice depend on the cognitive effort produced during retrieval attempts (Glover, 1989; Karpicke & Roediger, 2007; Pyc & Rawson, 2009). When retrieval becomes easier and the level of effort declines, the advantage of retrieval practice is expected to diminish. Increased learning rounds typically improve retrieval success, particularly with corrective feedback, and reduce the cognitive effort required for subsequent retrievals, but the current study did not directly manipulate or measure retrieval difficulty in the intervention phase. This limitation prevents us from determining whether retrieval effort remained stable throughout learning. Alternatively, these results could be interpreted as suggesting that the reduction in retrieval effort across learning rounds may not have reached the threshold required to attenuate the benefits of retrieval practice. This interpretation is consistent with Nakata’s (2017) finding that repeated retrieval within a single session continued to enhance memory even after seven times, indicating that retrieval effort had not yet declined to a level that would attenuate its benefits. In comparison, learners in the present study only engaged in up to four rounds of retrieval, suggesting that the number of repetitions may have been insufficient to cause a significant drop in retrieval effort.

The current findings’ misalignment with the predictions of the retrieval effort hypothesis may have been moderated by the use of corrective feedback, since such feedback supports immediate error correction, thereby strengthening the re-encoding of accurate information and reducing interference from false memories (Butler & Roediger, 2008; Kang et al., 2007). On one hand, its verification function helps reduce learners’ uncertainty, raising retrieval success and lessening the cognitive load of each attempt. On the other hand, it introduces additional encoding opportunities to reinforce semantic connections. Although feedback may lower retrieval effort, it simultaneously activates alternative enhancement pathways via error correction and semantic elaboration. Consequently, the results observed in the current study may indicate that the benefits of these additional processes were sufficient to maintain, and even enhance, the advantages of retrieval-based learning.

In addition, the present study shows no significant differences in learners’ performance across different test modalities, indicating that variations in testing context do not affect the effectiveness of either digital flashcard or word-list learning. This result is consistent with Halamish and Elias (2022), both in terms of the nonsignificant effect of testing media and the consistent advantage of flashcard over word-list learning. In addition, the present study reveals that with an increased number of learning rounds, the effect of test modality remains nonsignificant, suggesting that learning opportunities has not significant impact on the efficacy of learning methods across different test modalities. Taken together, these findings suggest that digital flashcard learning is a stable and reliable learning method whose advantages remain consistent across different testing contexts.

On the basis of the contextual variability hypothesis (Lohnas et al., 2011), retrieval practice and restudy may induce different degrees of contextual variability in memory encoding. The dynamic nature of retrieval practice requires the reconstruction of target information within distinct cognitive contexts during each attempt, thereby facilitating the formation of more generalized and context-independent memory representations. In contrast, restudying predominantly involves repetitive exposure to the learning context, resulting in memory traces that remain tightly bound to their initial encoding conditions. Previous research has demonstrated that knowledge acquired through retrieval practice adapts more effectively to novel contexts, providing empirical support for this theoretical framework (Butler, 2010; Karpicke & Blunt, 2011; Rohrer et al., 2010). However, the expected results were not observed in this study when the test modality was manipulated.

A plausible explanation for this outcome is the limited external contextual information available in the experimental design. In the current study, the learning materials consisted of Swahili–Chinese word pairs, providing only orthographic and semantic information. Furthermore, these materials were presented in a uniform visual format, with identical font, size, color, and on-screen location, across two learning methods, creating a virtually identical external learning environment. In contrast, prior studies employed more complex materials such as educational texts or factual knowledge, which introduced diverse external contexts across learning conditions, thus allowing for greater opportunity for contextual variation. For instance, in the study by Rohrer et al. (2010), students learned city locations on a map through retrieval practice (matching names to locations), which subsequently improved their performance on a transfer test that required recalling the spatial locations of cities along routes connecting other cities.

Another plausible explanation for this outcome is that changing the testing modality may not sufficiently engage the deeper cognitive processes necessary to reveal memory flexibility. To investigate the flexibility of memory formed through different learning methods, previous studies have often varied the test format. Two main approaches have been commonly used. The first involves changing the type of questions between the learning and testing phases. For example, Hinze and Wiley (2011, Experiment 3) required participants to complete fill-in-the-blank or paragraph recall tasks during learning but administered multiple-choice questions in the final test. Their results demonstrated that retrieval practice facilitated knowledge transfer. The second approach maintains the question type constant while modifying the specific wording of the test items. For instance, McDaniel et al. (2007) used fill-in-the-blank questions in both phases but varied the omitted words and consistently observed robust retrieval practice effects. Such manipulations introduce substantive variations in the retrieval context and engaged deeper cognitive processing, thereby providing a more sensitive measure of memory flexibility and context dependence. In contrast, the present study varied only the test modalities (handwriting vs. typing) while keeping the task structure and question content identical. From a context-dependent memory perspective, this represents a relatively superficial change that does not substantially alter the retrieval environment. As a result, differences in context dependence or memory flexibility between retrieval practice and restudy may not be detectable under such conditions.

## 6. Educational Implications

Based on the present findings, DFs demonstrate a clear advantage in FL vocabulary learning. Retrieval-based learning with DFs consistently outperformed restudy using word lists under all learning and testing conditions, indicating that vocabulary instruction should prioritize active recall and corrective feedback rather than passive review. Moreover, the learning advantage of DFs increases with the number of learning rounds, suggesting that repeated retrieval has a cumulative effect in reinforcing memory representations. Therefore, instructional design should incorporate multiple retrieval opportunities within learning sessions, enabling learners to consolidate knowledge progressively.

No significant interaction is observed between learning method and test modality, indicating that the benefit of retrieval practice is robust across different testing contexts. This finding suggests that DFs can be flexibly used across different learning environments and devices, making them suitable for blended or mobile learning contexts. Learners can review and self-test on digital devices without compromising learning outcomes. Overall, these results indicate that DFs provide an efficient and stable tool for promoting FL vocabulary learning.

## 7. Limitations and Future Research

The present study found that the RPE of DFs increased with the number of learning rounds. However, it remains unclear what specific number of rounds is needed to achieve optimal learning outcomes. Previous research has indicated that five to seven retrieval repetitions produce superior learning outcomes compared to one to three repetitions (Nakata, 2017). These findings suggest that future studies should include higher numbers of retrieval attempts to systematically determine the point at which additional repetitions no longer yield incremental benefits to the retrieval practice effect.

In addition, although the study adopted a relatively long retrieval interval of six minutes, multiple learning rounds were completed within a relatively short timeframe of approximately 30 min. This condensed schedule differs substantially from the distributed learning patterns characteristic of authentic language contexts, which unfold over weeks or semesters, thereby limiting the generalizability of the findings to long-term learning scenarios. To enhance ecological validity and practical relevance, future research should apply extended designs with retrieval intervals. Establishing a temporal framework that more closely mirrors authentic learning environments would allow researchers to track the cumulative effects of retrieval practice on long-term vocabulary acquisition and generate more generalizable evidence for optimizing language instruction.

Another limitation of the current study is retrieval effort not being directly monitored during the learning phase. Although retrieval practice demonstrates a clear advantage, cognitive load is indirectly inferred from the number of learning rounds, leaving it unclear whether participants’ retrieval effort remains stable or declines across rounds. Future studies should measure retrieval effort during the intervention phase, using indicators such as subjective difficulty ratings or response times to clarify its role in modulating learning outcomes.

Finally, the manipulation of external context by changing test modality represented a surface-level adjustment and may have been insufficient to reveal differences in memory flexibility between retrieval practice and restudying. Future research could introduce more cognitively meaningful variations in test formats, such as by systematically manipulating retrieval direction following Carpenter et al. (2006) to require the retrieval of both receptive (recognizing word forms/meanings) and productive knowledge (producing word forms/meanings) across learning and testing phases. Such design would provide a more sensitive measure of context-dependent memory and illuminate how memory representations are modulated by different learning methods.

## Figures and Tables

**Table 1:** Scores of participants’ L2 proficiency (M±SD).

	Self-Assessment Score				LexTale Score * (%)
	Listening	Speaking	Reading	Writing	
3-round	3.44 ± 1.06	3.26 ± 1.15	4.67 ± 0.95	3.72 ± 0.90	0.56 ± 0.07
4-round	3.48 ± 1.04	3.54 ± 1.16	4.59 ± 1.07	3.89 ± 1.06	0.57 ± 0.08

Note *: Since five participants did not complete the Lextale test, the final sample size included in the statistical analysis was 103. The correspondence between score of LexTale and the CEFR proficiency levels were: ≤59% = B1 level; 60–80% = B2 level.

**Table 2:** Stroke number of Chinese translations and recall accuracy across of Swahili–English word pair after three cycles of learning and testing (M±SD).

	Sublist 1	Sublist 2	Sublist 3	Sublist 4
Stroke Number	17.00 ± 6.20	15.87 ± 4.47	16.73 ± 6.20	16.73 ± 3.99
Recall Accuracy	0.59 ± 0.14	0.63 ± 0.13	0.63 ± 0.12	0.61 ± 0.11

**Table 3:** Descriptive statistics for recall accuracy across conditions (*M* ± *SD*).

	RSP	RTP	RSC	RTC
3-round	0.39 ± 0.24	0.48 ± 0.25	0.42 ± 0.26	0.50 ± 0.25
4-round	0.51 ± 0.27	0.65 ± 0.26	0.50 ± 0.25	0.65 ± 0.24

## Data Availability

The datasets used during the current study are available from the corresponding author on reasonable request.

## Supplementary Materials

The following supporting information can be downloaded at: https://www.mdpi.com/article/10.3390/bs15111540/s1. S1: Pre-experiment survey; S2: Learning materials; S3: Counterbalancing design of learning materials.
