# Supplementary material for: The Moderating Role of Learning Rounds: Effects on Retrieval Practice and Context Dependence in Digital Flashcard Foreign Language Vocabulary Learning
Source: Behav Sci (Basel). 2025 Nov 12;15(11):1540. doi: 10.3390/bs15111540 (PMC12649105; doi:10.3390/bs15111540)
Supplement: Supplementary file 1 [file behavsci-15-01540-s001.zip › behavsci-3941710-supplementary.pdf]

Supplementary Information S1. Pre-experiment survey.

1. How difficult do you think it is to memorize vocabulary in a foreign language?
   - ☐ 1 = very easy
   - ☐ 2 = easy
   - ☐ 3 = relatively easy
   - ☐ 4 = moderate
   - ☐ 5 = relatively difficult
   - ☐ 6 = difficult
   - ☐ 7 = very difficult
2. Have you ever used electronic media (such as using digital vocabulary books or flashcard applications) to study vocabulary in a foreign language?

   ☐ Yes ☐ NO
3. Have you ever used electronic media (such as online quizzes or computer-based assessments) to take vocabulary tests in a foreign language?

   ☐ Yes ☐ NO
4. Do you think that different test media (such as paper-based test vs. computer-based test) will affect your performance when you take foreign vocabulary test?

   ☐ Yes ☐ NO

Supplementary Information S2. Learning materials.

Sublist 1		
**Swahili**	**English**	**Chinese**
ambo	glue	胶水
goti	knee	膝盖
ankra	invoice	发票
maiti	corpse	尸体
chama	society	社会
fumbo	mystery	奥秘
godoro	mattress	床垫
mshoni	tailor	裁缝
nafaka	corn	谷物
lawama	blame	责备
kasuku	parrot	鹦鹉
malkia	queen	王后
fununu	rumor	谣言
baharia	sailor	水手
bustani	garden	花园

Sublist 2		
**Swahili**	**English**	**Chinese**
lozi	almond	杏仁
sumu	poison	毒药
fagio	broom	扫把
elimu	science	科学
duara	wheel	轮子
vumbi	dust	灰尘
adhama	honor	荣誉
rushwa	bribe	贿赂
mashua	boat	轮船
nyanya	tomato	番茄
fahali	bull	公牛
hariri	silk	丝绸
zabibu	grapes	葡萄
desturi	custom	风俗
tumbili	monkey	猴子

(Continued)

Sublist 3		
**Swahili**	**English**	**Chinese**
jani	leaf	叶子
ndoo	bucket	水桶
pombe	beer	啤酒
zulia	carpet	地毯
chura	frog	青蛙
mfupa	bone	骨头
hamira	yeast	酵母
yamini	oath	誓言
samadi	manure	肥料
jeraha	wound	伤口
farasi	horse	马匹
dafina	treasure	宝藏
yatima	orphan	孤儿
bahasha	envelope	信封
zeituni	olives	橄榄

Sublist 4		
**Swahili**	**English**	**Chinese**
ziwa	lake	湖泊
leso	scarf	围巾
pazia	curtain	窗帘
rembo	ornament	装饰
kamba	rope	绳子
ladha	flavor	味道
tajiri	merchant	商人
talaka	divorce	离婚
kaburi	grave	坟墓
utenzi	poem	诗歌
sahani	plate	盘子
tabibu	doctor	医生
rafiki	friend	朋友
bandari	harbor	港口
handaki	trench	沟渠

Supplementary Information S3. Counterbalancing design of learning materials.

Version	Restudy	Retrieval practice	Learning order	Paper-based test	Computer-based test	Testing order
1	SL1 & 2	SL3 & 4	random	SL1 & 3	SL2 & 4	Paper - computer
2	SL1 & 2	SL3 & 4	random	SL2 & 4	SL1 & 3	Paper - computer
3	SL1 & 2	SL3 & 4	random	SL1 & 3	SL2 & 4	Computer - paper
4	SL1 & 2	SL3 & 4	random	SL2 & 4	SL1 & 3	Computer - paper
5	SL3 & 4	SL1 & 2	random	SL1 & 3	SL2 & 4	Paper - computer
6	SL3 & 4	SL1 & 2	random	SL2 & 4	SL1 & 3	Paper - computer
7	SL3 & 4	SL1 & 2	random	SL1 & 3	SL2 & 4	Computer - paper
8	SL3 & 4	SL1 & 2	random	SL2 & 4	SL1 & 3	Computer - paper

Note: Participants received corresponding experimental versions in their participation sequence. “SL” refers to sublist in the Supplementary Information S2.
